# Complete Genome Sequence of *Lelliottia* Podophage phD2B

**DOI:** 10.1128/genomeA.01046-14

**Published:** 2014-11-26

**Authors:** Grzegorz Nowicki, Jakub Barylski, Natalia Kujawa, Anna Goździcka-Józefiak

**Affiliations:** Department of Molecular Virology, Institute of Experimental Biology, Adam Mickiewicz University, Poznań, Poland

## Abstract

The genus *Lelliottia* was recently created from the group of environmental gammaproteobacteria previously included in the genus *Enterobacter*. Here, we report the complete genome sequence of phD2B, the first (according to our current knowledge) known phage that infects bacterium from the taxon.

## GENOME ANNOUNCEMENT

The genus *Lelliottia* was proposed by Brady and coworkers in 2013. It currently comprises two species: *L. amnigena* and *L. nimipressuralis* (1). These motile, facultatively anaerobic rods were isolated from water, food products, and plant tissues (*L. nimipressuralis* is notable for causing wetwood disease of trees) (2, 3). Neither *L. amnigena* nor *L. nimipressuralis* is considered pathogenic for mammals, but a few cases of human infections were reported (4, 5). In this report, we describe the complete genome of the first-known phage that infects *Lelliottia* sp.

Bacteriophage phD2B was isolated from a sediment sample from Lake Góreckie, an eutrophic reservoir located in Wielkopolski National Park, Poland. It infects strain GL2 isolated from the same reservoir and was identified as *Lelliottia* sp. (based on complete sequences of 16S rDNA as well as genes encoding hsp 60 GroEL chaperonin, β-subunits of RNA polymerase, and DNA gyrase).

The genome was sequenced using the 454 method in Genomed SA (Warsaw, Poland) and assembled using Newbler version 2.5.3 (454 Life Sciences) and Geneious version 6.1 (6) (64.98-fold coverage). Protein coding genes were predicted using GeneMarkS (7) and Prodigal version 2.60 (8). Their functional annotation was performed based on BLASTx hits against the UniprotKB viruses database (9). To search for tRNA genes we used tRNAscan-SE version 1.21 (10). Mapping of pyrosequencing reads that allows determination of genome ends was performed with the Pause pipeline (https://cpt.tamu.edu/computer-resources/pause/).

phD2B is an SP6-like podophage. The complete genome of the phage is 44,366 bp in length with a G+C content of 51% and ends with direct repeats (262 bp). We identified 49 coding sequences, all on one strand.

The arrangement of the modules seems to roughly reflect the order of expression. The left end of the genome contains a group of genes involved in the takeover of a host metabolism (*S*-adenosyl-l-methionine hydrolase and RNA polymerase) followed by a cluster of CDSs associated with replication and recombination (HNH endonuclease, primase/helicase, DNA polymerase, exonuclease, homing nuclease, and ligase). The right half is occupied mostly by a morphogenesis module. The last 4 to 5 kb of the genome encode proteins involved in DNA packaging and lysis of the host cell (terminase subunits, holin, and M15 family lytic peptidase). Surprisingly, the gene for the large terminase subunit is split into two open reading frames, which together encode the full-length protein. This suggests that we deal with exons of the single gene (separated by the intron that contains gene for homing endonuclease). A similar layout is rather uncommon for *Autographivirinae* (we found it only in the genome of phage K1E) but was observed in unrelated phages, including myoviruses (11–13) and siphoviruses (14–17).

### Nucleotide sequence accession numbers.

The complete sequence of phD2B genome is available from GenBank under the accession number KM370384. Sequences from the host bacterium are stored in records KM458060, KM458061, KM458062, and KM458063.
